# fNIRS-based brain functional response to robot-assisted training for upper-limb in stroke patients with hemiplegia

**DOI:** 10.3389/fnagi.2022.1060734

**Published:** 2022-12-09

**Authors:** Congcong Huo, Zhifang Sun, Gongcheng Xu, Xinglou Li, Hui Xie, Ying Song, Zengyong Li, Yonghui Wang

**Affiliations:** ^1^Rehabilitation Center, Qilu Hospital of Shandong University, Jinan, Shandong, China; ^2^Beijing Advanced Innovation Centre for Biomedical Engineering, Key Laboratory for Biomechanics and Mechanobiology of Ministry of Education, School of Biological Science and Medical Engineering, Beihang University, Beijing, China; ^3^Beijing Key Laboratory of Rehabilitation Technical Aids for Old-Age Disability, National Research Center for Rehabilitation Technical Aids, Beijing, China; ^4^Key Laboratory of Rehabilitation Aids Technology and System of the Ministry of Civil Affairs, Beijing, China

**Keywords:** stroke, motor rehabilitation, functional near-infrared spectroscopy, functional connectivity, cortical reorganization

## Abstract

**Background:**

Robot-assisted therapy (RAT) has received considerable attention in stroke motor rehabilitation. Characteristics of brain functional response associated with RAT would provide a theoretical basis for choosing the appropriate protocol for a patient. However, the cortical response induced by RAT remains to be fully elucidated due to the lack of dynamic brain functional assessment tools.

**Objective:**

To guide the implementation of clinical therapy, this study focused on the brain functional responses induced by RAT in patients with different degrees of motor impairment.

**Methods:**

A total of 32 stroke patients were classified into a low score group (severe impairment, *n* = 16) and a high score group (moderate impairment, *n* = 16) according to the motor function of the upper limb and then underwent RAT training in assistive mode with simultaneous cerebral haemodynamic measurement by functional near-infrared spectroscopy (fNIRS). Functional connectivity (FC) and the hemisphere autonomy index (HAI) were calculated based on the wavelet phase coherence among fNIRS signals covering bilateral prefrontal, motor and occipital areas.

**Results:**

Specific cortical network response related to RAT was observed in patients with unilateral moderate-to-severe motor deficits in the subacute stage. Compared with patients with moderate dysfunction, patients with severe impairment showed a wide range of significant FC responses in the bilateral hemispheres induced by RAT with the assistive mode, especially task-related involvement of ipsilesional supplementary motor areas.

**Conclusion:**

Under assisted mode, RAT-related extensive cortical response in patients with severe dysfunction might contribute to brain functional organization during motor performance, which is considered the basic neural substrate of motor-related processes. In contrast, the limited cortical response related to RAT in patients with moderate dysfunction may indicate that the training intensity needs to be adjusted in time according to the brain functional state. fNIRS-based assessment of brain functional response assumes great importance for the customization of an appropriate protocol training in the clinical practice.

## Introduction

The recovery of upper-limb motor function is still limited in stroke survivors, which significantly impacts their independence of daily living (Lawrence et al., 2001; Stoykov et al., 2009). Recently, robot-assisted therapy (RAT) for the upper-limb has emerged as a popular rehabilitation intervention for stroke. Several studies have verified the clinical effectiveness of RAT based on clinical assessment (Sale et al., 2014) and biomechanical parameters, including kinematic and kinetic parameters (Mazzoleni et al., 2011, 2013). Besides, recent reviews have reported heterogeneous outcomes of RAT among stroke patients (Veerbeek et al., 2017; Mehrholz et al., 2018), mainly because the severity of hemiparesis was not considered as an important feature when choosing an appropriate pattern of RAT for a patient. There is a lack of clinically effective assessment tools to assist the therapist to deliver appropriate therapeutic interventions according to the specific need of each patient, especially for the patients with moderate to severe hemiplegia. Plastic reorganization of the brain is essential for functional recovery after stroke (Cirillo et al., 2020). As regard this issue, it is necessary to evaluate the specific functional response patterns associated with RAT in stroke patients with different degree of motor impairment. Real-time characterization of the brain functional responses to specific interventions assumes great importance for the customization of an appropriate protocol training as to reach substantial improvement in the clinical practice.

The real-time monitoring of cortical responses during motor intervention still remains challenging due to the low tolerance of motion artifact of some imaging techniques, such as functional magnetic resonance imaging (fMRI) and electroencephalography (EEG). Functional near-infrared spectroscopy (fNIRS) is an emerging noninvasive method that monitors cortical haemodynamics with advantages including safety, portability, and motion artifact tolerance (Murata et al., 2002, 2006). In recent years, significant progress made in fNIRS technology make it attract considerable attention in various research and clinical settings (Hong and Yaqub, 2019). Brain functional features collected based on fNIRS can be used as a biomarker of mild cognitive impairment (MCI)(Yang et al., 2020). fNIRS can also be used to evaluate the characteristics of brain function response induced by cognitive interventions such as acupuncture therapy in patients with MCI (Ghafoor et al., 2019). In addition, several fNIRS studies have demonstrated the feasibility of fNIRS for brain-computer interface (BCI), which can be widely used in the field of neurorehabilitation, motor rehabilitation and entertainment, etc. (Naseer and Hong, 2015). The combination of fNIRS, EEG, and other technologies can improve the classification accuracy of BCI system by decoding brain activities under multimodal neuroimaging modalities (Hong and Khan, 2017). Based on the principle of optical imaging, fNIRS can be utilized in combination with electromagnetic neuromodulation technologies (such as transcranial electrical stimulation) to monitor the cortical response and provide real-time feedback for these interventions (Yang et al., 2021). The application of fNIRS can not only get insight into the mechanism underlying neuromodulation in neurorehabilitation, but also provide targeted neuromodulation based on closed-loop regulation to achieve personalized therapy for brain disorders (Hong et al., 2022). Additionally, a comprehensive review described fNIRS-related applications for stroke and suggested fNIRS as a promising technology for detecting brain function responses during specific rehabilitation interventions (Saitou et al., 2000). Using fNIRS, we previously reported the involvement of the prefrontal, motor and occipital areas during limb-linkage training (Huo et al., 2019) and unilateral/bilateral upper limb training in stroke patients (Xu et al., 2022). The prefrontal cortex (PFC) contributes to attention, planning, decision-making and the synthesis of diverse information needed for goal-directed behaviour (D’Esposito et al., 2000; Miller and Cohen, 2001). Motor-related areas are mainly involved in the coordination and execution of sensory and motor functions for complex movements (Moran and Desimone, 1985; Peterka and Loughlin, 2004). The occipital lobe (OL) is crucial for the conscious perception of body parts and can be modulated by visual stimuli, visual-guided attention and motor action (Miller et al., 1993; Astafiev et al., 2004). Functional connectivity (FC) analysis is commonly used to explore the neural interactions within the brain functional network, providing insight into the understanding of cortical reorganization and behaviour deficits after stroke (Friston, 2011; Corbetta et al., 2018). It is speculated that these regions may be involved in a specific coordinated pattern in response to motor therapy for stroke rehabilitation.

At such, the primary aim of this study was to evaluate the specific cortical network response patterns to RAT based on fNIRS in combination with FC analysis in subacute stroke patients with unilateral moderate-to-severe motor deficits, supporting the hypothesis of different functional response patterns associated with RAT depend on the degree of impairment. Real-time assessment of brain functional response can provide a theoretical basis for choosing the appropriate protocol for a patient to support the clinical decision.

## Materials and methods

### Participants

**Figure 1:**
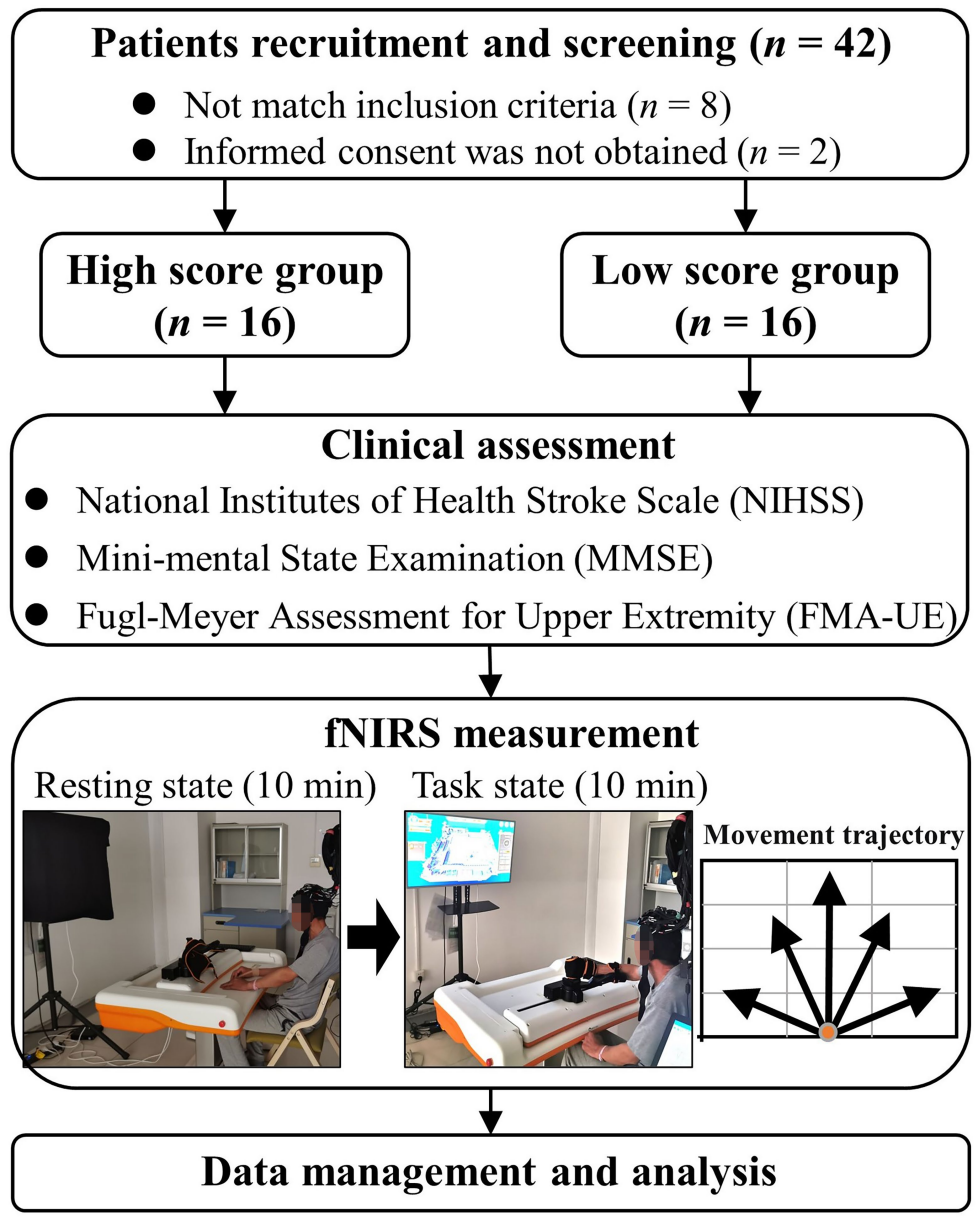
Flow-chart through this study.

The flow-chart through this study is shown in Figure 1. Thirty-two first-ever patients with stroke hemiplegic participated in this study. All subjects were right-handed according to the Chinese edition of the Handedness Inventory (Oldfield, 1971). Inclusion criteria: (Stoykov et al., 2009) unilateral lesions; (Lawrence et al., 2001) moderate to severe motor impairment of the hemiplegic upper limb; (Sale et al., 2014) ability to understand and follow experimental tasks; (Mazzoleni et al., 2013) age between 18 and 80 years. Exclusion criteria: (Stoykov et al., 2009) clinically unstable medical disorders; (Lawrence et al., 2001) severe cognitive impairment. The baseline characteristics (age, sex, time post stroke, lesion location) and clinical assessments, including the National Institutes of Health Stroke Scale (NIHSS), Mini-mental State Examination (MMSE) and Fugl-Meyer assessment for upper-extremity (FMA-UE), were assessed for each patient (Table 1). Of note, patients were classified into 2 groups (those with a low score reflecting severe impairment and those with a high score reflecting moderate impairment) by the median FMA-UE score (median: 18) to investigate the effects of motor impairment on the brain functional response to RAT.

**Table 1:** Clinical characteristics of stroke patients.

**No.**	**Age/Sex**	**Duration (days)**	**Type**	**NIHSS**	**FMA-UE**	**MMSE**	**Lesion site**
1	56/M	43	Hemorrhage	8	13	26	L-BG
2	73/M	21	Infarction	4	14	28	L-BG, CR
3	49/M	152	Hemorrhage	9	6	26	L-BG, CR
4	34/F	112	Hemorrhage	9	19	25	L-BG
5	52/M	53	Infarction	3	16	28	L-BG, CR
6	34/F	123	Hemorrhage	9	27	26	L-BG
7	29/M	65	Infarction	3	33	28	R-BG
8	50/M	64	Hemorrhage	7	17	29	L-BG, CR
9	49/M	138	Hemorrhage	9	7	28	L-BG, CR
10	53/M	177	Infarction	5	20	26	L-BG
11	29/M	50	Infarction	3	31	27	R-BG
12	66/F	141	Infarction	3	35	28	R-BG, CR
13	34/F	132	Hemorrhage	9	28	27	L-BG
14	46/M	177	Hemorrhage	10	16	28	L-BG, CR
15	53/M	57	Infarction	5	15	25	L-BG
16	37/M	145	Hemorrhage	2	46	27	R-BG
17	66/F	126	Infarction	3	33	28	R-BG, CR
18	29/M	84	Infarction	3	36	29	R-BG
19	55/F	34	Hemorrhage	8	16	27	R-BG
20	37/M	121	Hemorrhage	2	44	27	R-BG
21	66/F	161	Infarction	3	43	28	R-BG, CR
22	50/F	59	Hemorrhage	11	10	27	R-BG
23	38/M	78	Hemorrhage	10	8	25	R-BG
24	50/F	20	Infarction	5	35	29	R-BG
25	57/F	35	Hemorrhage	3	38	27	R- BG
26	50/M	173	Hemorrhage	7	16	28	R-BG
27	64/F	72	Infarction	6	14	28	R-BG
28	73/M	127	Infarction	3	19	27	L-BG, CR
29	52/M	39	Infarction	3	14	29	L-BG, CR
30	52/M	61	Infarction	3	15	26	L-BG, CR
31	50/M	43	Hemorrhage	7	4	27	L-BG, CR
32	37/M	135	Hemorrhage	2	46	27	R-BG

F: Female; M: Male; L: Left; R: Right; BG: Basal ganglia; CR: Corona radiata. NIHSS: National Institutes of Health Stroke Scale; MMSE: Mini-mental State Examination; FMA-UE: Fugl-Meyer assessment for upper-extremity.

Experiments were conducted with the understanding and written consent of each patient or the family members. The experimental study (Trial Registration: ChiCTR2100048433) was approved by the Medical Ethics Committee of Qilu Hospital and carried out according to the ethical standards defined by the Helsinki Declaration in 1975 (revised in 2008).

### RAT task and fNIRS data acquisition

During the experiment, patients were asked to undergo data acquisition in the sitting position in both the resting state (10 min) and task state (10 min). During the resting state, patients were asked to remain still and relax with their eyes closed but stay awake. The robotic system (Arm Motus™, Shanghai Fourier Intelligence Technology Co., Ltd., China) designed for clinical rehabilitation applications was used for this study. During the task state, the hemiparetic forearm of patients was positioned on the robot-assisted upper-limb training instrument (end effector type) with an arm bracket secured to the forearm and a handle was fixed to the affected hand with bandages. The robotic system provides the goal-directed and planar reaching movements of shoulder and elbow through an “assisted as needed” control strategy at an equally speed (5.0 cm/s) and range of motion (medium: Y-axis = 20 cm, X-axis = 30 cm) around a centre target. The Movement trajectory and space is shown in Figure 1. During the task state, the patients were requested to avoid any movements other than those needed for motor tasks. A professional therapist was involved in the whole experiment to ensure the safety of the participants.

**Figure 2:**
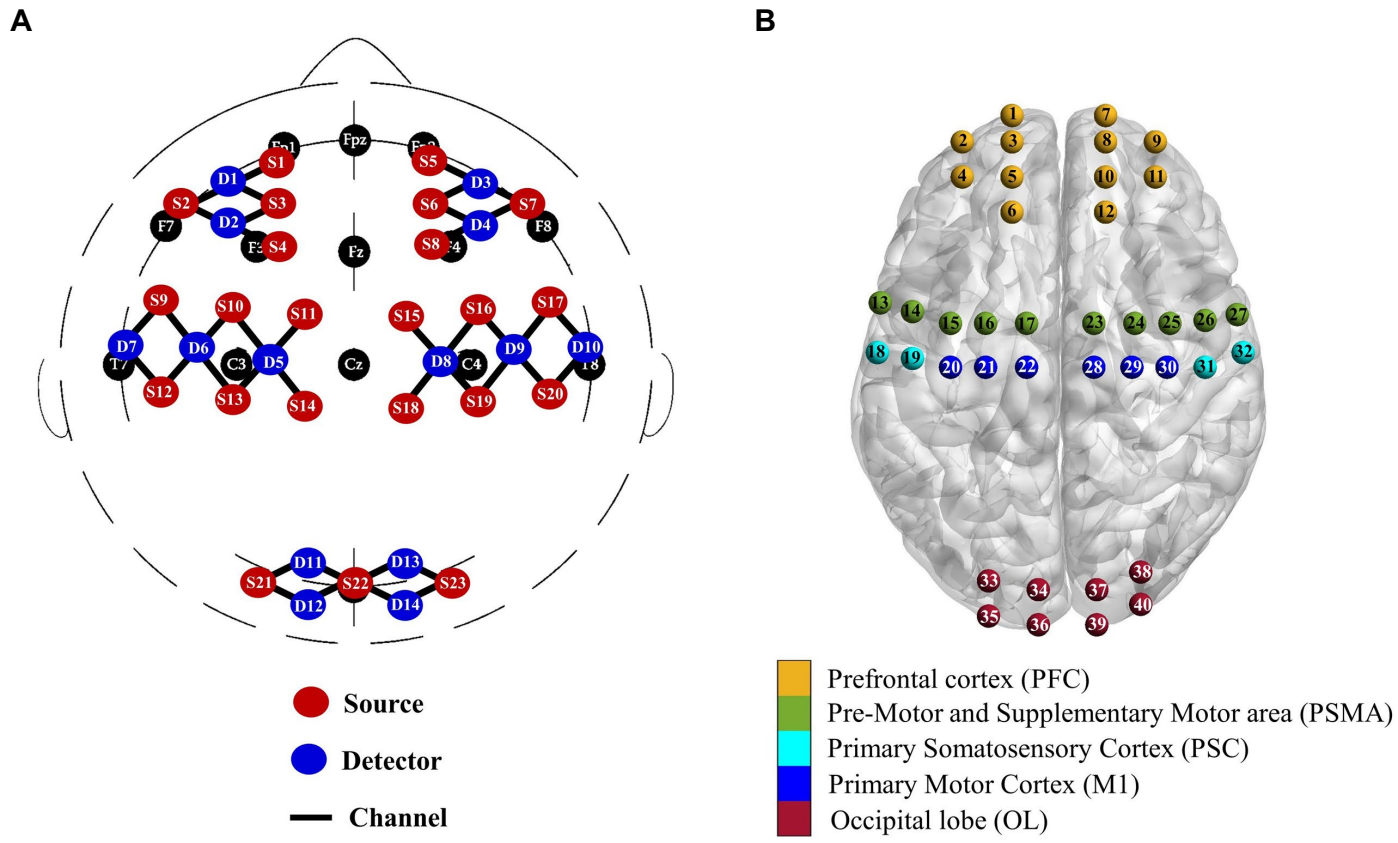
Multichannel fNIRS configurations in international 10–10 system **(A)** and corresponding brain regions of interest **(B)**.

During each session, cerebral haemodynamics were continuously monitored using a continuous-wave fNIRS device (Nirsmart, Danyang Huichuang Medical Equipment Co, Ltd., China) with 23 sources and 14 detectors at a sampling rate of 10 Hz. The differential path-length factors (DPFs) were set to 6. A total of 40 channels were positioned over the left and right PFC (symmetric with FpZ as a reference), motor cortex (corresponding areas C3 and C4) and OL (symmetric with OZ as a reference) according to the international 10–10 system of electrode placement (Figure 2A). The interoptode distance was 30 mm. As shown in Figure 2B, the regions of interest (ROIs), including the bilateral PFC, primary motor cortex (M1), primary somatosensory cortex (PSC), premotor and supplementary motor area (PSMA), and OL, were defined based on fNIRS channel locations recorded by 3D digitization. For patients with lesions on the right, the lesion side was uniformly set to the left hemisphere by flipping the fNIRS channels from right to left about the midsagittal line for patients with lesions on the right. In this study, the ROIs in the ipsilesional and contralesional hemispheres were presented as i-PFC, i-PSC, i-PSMA, i-M1, i-OL, and c-PFC, c-PSC, c-PSMA, c-M1, c-OL, respectively.

### fNIRS data preprocessing

For fNIRS data, first, the absorbance signals recorded by fNIRS were bandpass filtered at 0.0095–2 Hz (zero-phase, fifth-order Butterworth filter) to reduce the uncorrelated noise components and low-frequency baseline drift. Then, fluctuations in the concentration of oxygenated haemoglobin (delta HbO_2_) were calculated from the filtered light density according to the modified Beer–Lambert law (Cope and Delpy, 1988). The first 1 min of delta HbO_2_ data were excluded to reach a steady state, with 5,400 remaining time points for each patient. We then applied principal and independent component analysis to reduce physiological interference in fNIRS measurements to extract the functional response in the brain (Santosa et al., 2013). The components of interest were visually identified according to the criteria that the relevant time course has a remarkable low-frequency spectrum (0.01–0.08 Hz) for functional haemodynamic responses (Cordes et al., 2001). This study focuses on delta HbO2 signal for subsequent analysis, mainly delta HbO2 data has better signal to noise ratio and a stronger correlation with blood-oxygenation level-dependent signal measured by fMRI (Anwar et al., 2013; Visani et al., 2015). Data preprocessing including motion artifact removal was described in our previous study (Huo et al., 2019).

### FC analysis based on wavelet phase coherence

Wavelet transforms have the ability to decouple signal components and provide localized phase information. With the complex Morlet wavelet, the wavelet coefficients are complex numbers and can define the instantaneous relative phase information for each frequency and time. Wavelet transforms can be used to examine the relationship among oscillations (Li et al., 2014). FC can be calculated based on the wavelet phase coherence (WPCO) index to describe the statistical interdependencies between two haemoglobin oscillatory components by examining how phase differences align within a specific frequency range (Bandrivskyy et al., 2004; Bernjak et al., 2012). The amplitude-adjusted Fourier transform (AAFT) surrogate test was used to confirm whether the detected coherence parameters were genuine or spurious (Stankovski et al., 2017). Tan et al. described the calculation procedure for the WPCO and AAFT tests in detail (Tan et al., 2015). In this study, oscillators of delta HbO_2_ signals in 0.01–0.08 Hz were identified using wavelet transform. Based on the AAFT test, significant channel-wise FC was obtained for each channel pair among the fNIRS oscillations for each condition.

#### Interregional and intraregional FC analysis

Based on the significant channel-wise FC matrix for each condition, we calculated the interregional and intraregional FC of the ROIs to analyse task-related changes in the large-scale network. The interregional FC among ROIs was calculated by averaging the WPCO values across all involved channel-wise connection edges based on the fNIRS channel distribution, generating a 10×10 region-wise FC matrix. The intraregional FC was calculated by averaging the WPCO values of the involved channel-wise connection edges within each of the ROIs, generating 10 intraregional FC values.

#### Correlation analysis of task-related FC changes and clinical variables

To identify the relationship between the brain functional response related to the RAT and the clinical functional status of the upper-limb, partial correlations were employed to assess the correlation between task-induced changes in FC (delta FC = $\;{\mathrm{FC}}_{task}-{\mathrm{FC}}_{rest}$ ) and the FMA-UE score with age and time poststroke as nuisance regressors for each group of stroke patients.

#### Brain lateralization analysis based on the hemisphere autonomy index

The connection-based hemispheric autonomy index (HAI) was calculated for each significant FC matrix to further describe the functional network architecture of specific states for stroke patients. Based on the significant channel-wise FC matrix, the HAI was calculated according to the definition:


$$\mathrm{AI}\;=\frac{\mathrm{Ni}\_m}{\mathrm{Ti}\_m}-\frac{\mathrm{Nc}\_m}{\mathrm{Tc}\_m.}$$

where *m* represents any fNIRS channel (*m* = 1,2, ⋯ 40); Ni_m and Nc_m are the number of channels connected to channel *m* within (ipsilateral) hemisphere and between (contralateral) hemisphere, respectively; and Ti_m and Tc_m represent the total number of channels connected with channel m in the ipsilateral and contralateral hemispheres, respectively. The HAI is calculated for each channel as the index describing brain lateralization based on the difference between intrahemispheric and interhemispheric connectivity with each channel. This approach yielded HAI values that ranged between −1 and 1. A higher HAI value indicated more intrahemispheric connectivities than interhemispheric connectivities.

### Statistical analysis

In this study, we used the G-power (v3.1.9.2; Franz Faul, University of Kiel, Kiel, Germany) for calculation of the sample size based on a previous fNIRS study that investigated the functional network patterns of stroke patients related to rehabilitation training (Lu et al., 2019). We set the effect size as 0.52, an α -error of 0.05 and a β of 0.20 (power level of 0.80). According to the analysis, at least 32 patients were needed in order to make an adequate group size, thus a sample size of 16 per group. The Kolmogorov–Smirnov test was used to determine whether values for the assessments were normally distributed. Demographic data including sex and type of stroke was compared by groups using a chi-square test. Age, duration of stroke, and functional assessment (MMSE, NIHSS, BI, and FMA-UE) were compared using one-way ANOVA. Based on this group classification, significant within-group and between-group differences in connection related indices (channel-wise FC, interregional FC, and intraregional FC) were evaluated using repeated–measures ANOVA and *post hoc t*-test with false discovery rate (FDR)-corrected for multiple comparisons. The association of upper-limb functional status and cortical response was examined by correlating FMA-UE with FC changes related to RAT with age and time post stroke as nuisance regressors. The Mann–Whitney U test was used to analyse the within-group and between-group differences in the HAI obtained from each condition. Statistical significance was set at *p* < 0.05.

## Results

### Demographic information

All 32 participants completed the study. Patients were classified as having moderate (high score group, *n* = 16, FMA-UE: 33.31 ± 9.04) or severe (low score group, *n* = 16, FMA-UE: 12.56 ± 4.16) upper-limb motor impairment according to the median of the FMA-UE. No significant between-group differences were noted in the characteristics of patients, including age, sex, time poststroke, stroke type and MMSE (*p* > 0.05, see Table 2). One-way ANOVA showed that the NIHSS score of patients in the low score group significantly higher than that of patients in the high score group (*p* = 0.006).

**Table 2:** Comparison of basic information of two groups of patients.

Characteristics	High score group (*n* = 16)	Low score group (*n* = 16)	Statistical result
Sex (M/F)	8/8	13/3	$\chi^{2}=$ 3.463, *p* = 0.063
Age (years)	45.69 ± 15.61	52.43 ± 7.63	*F* = 2.414, *p* = 0.131
Time post-stroke (months)	3.65 ± 1.52	2.65 ± 1.74	*F* = 2.991, *p* = 0.094
NIHSS	4.19 ± 2.54	6.88 ± 2.66	*F* = 8.573, *p* = 0.006*
FMA-UE	33.31 ± 9.04	12.56 ± 4.16	*F* = 1.405, *p* = 0.242
MMSE	27.25 ± 1.06	27.19 ± 1.28	*F* = 0.023, *p* = 0.881

Data are presented as mean ± SD. *p < 0.05.

### Effects of motor impairment on RAT-related changes in FC

**Figure 3:**
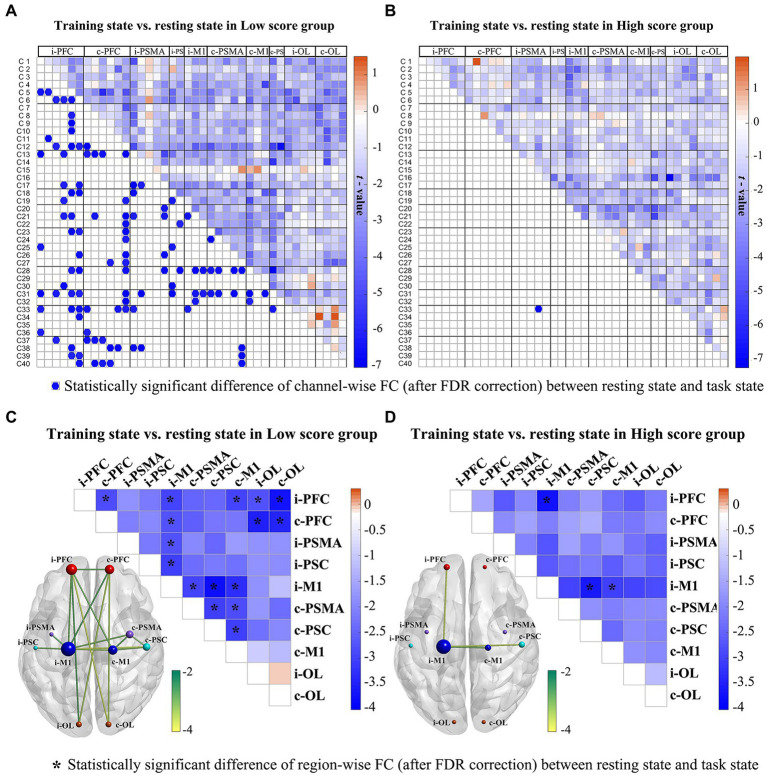
Changes in channel-wise FC **(A, B)** and region-wise FC **(C, D)** in response to RAT for the low score and high score groups. In the first row, the upper triangle represents the *t*-value of channel-wise FC between the two states, while the blue dot on the bottom triangle represents the statistically significant difference of channel-wise FC after FDR correction between the two states in each stroke group. The second row displays the *t*-values of region-wise FC between the two states. The *represents the statistically significant difference of region-wise FC after FDR correction between the two states in each stroke group. The size of a node indicates how many significant edges are connected to this region. Different node colours represent different brain regions. *FDR-corrected *p* < 0.05.

For within-group statistical result of channel-wise FC, the low-score group showed significantly decreased WPCO values in the RAT state compared to the resting state (Figure 3A), which were mainly distributed between the prefrontal and motor areas, between the prefrontal and occipital brain areas, and between the bilateral motor related brain areas. For the high-score group (Figure 3B), only one channel-wise WPCO value (between Ch. 33 and Ch. 16) was significantly decreased in the task state compared with the resting state, which were significant at strict FDR-corrected thresholds. For the large-scale interregional and intraregional FC, task-state FC correlation strengths were consistently lower than resting-state FC strengths among cortical regions in both groups of patients. The results showed that in the low score group, during the task state compared with the resting state, significantly decreased interregional FC values were observed in the network (Figure 3C), including connectivities between the i-PFC and c-PFC (*t* = 2.845; *p* = 0.012), i-M1 (*t =* 2.975*; p =* 0.009), c-M1 (*t* = 2.957; *p* = 0.010), i-OL (*t* = 2.740; *p* = 0.015), c-OL (*t* = 3.730; *p* = 0.002), and between the c-PFC and i-M1 (*t* = 2.731; *p* = 0.015), i-OL (*t* = 3.474; *p* = 0.003), c-OL (*t* = 3.424; *p* = 0.004), and between the i-M1 and i-PSMA (*t* = 2.763; *p* = 0.015), i-PSC (*t* = 3.000; *p* = 0.009), c-PSMA (*t* = 2.987; *p* = 0.009), c-PSC (*t* = 2.755; *p* = 0.002), c-M1 (*t* = 3.281; *p* = 0.005), and between the c-M1 and c-PSMA (*t* = 2.820; *p* = 0.013), c-PSC – c-M1 (*t* = 3.034; *p* = 0.008), and between the c-PSMA – c-PSC (*t* = 3.011; *p* = 0.009). For the high score group (Figure 3D), the interregional FC of connectivities between the i-M1 and i-PFC (*t* = 3.697; *p* = 0.002), c-PSC (*t* = 3.583; *p* = 0.003), c-M1 (*t* = 2.980; *p* = 0.009) was significantly decreased in the task state compared with that in the resting state. There were no significant differences between groups after FDR correction.

**Figure 4:**
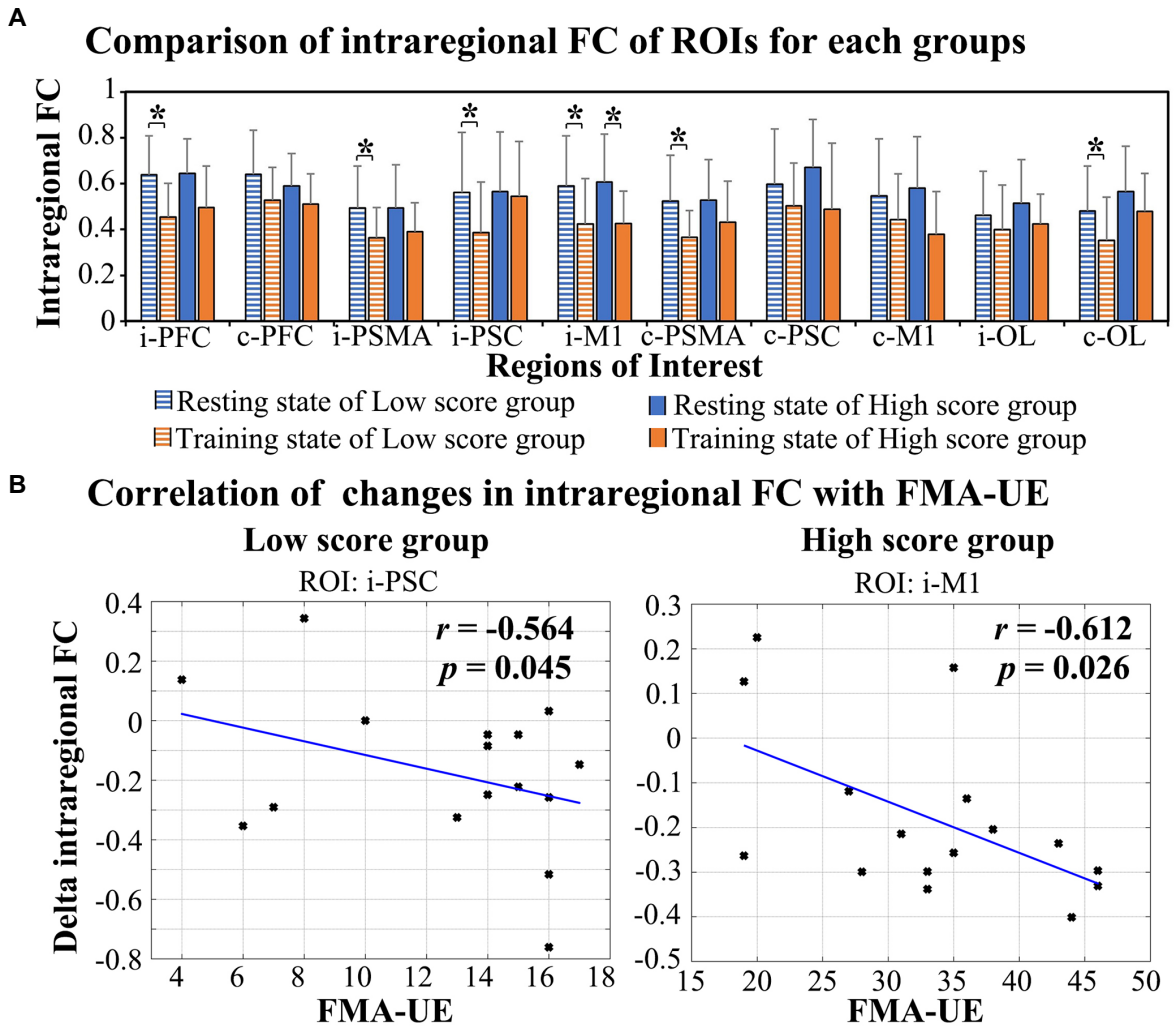
Alterations in intraregional FC in response to RAT in the two groups **(A)** and the relationship between the delta FC and FMA-UE score **(B)**. *FDR-corrected *p* < 0.05.

As shown in Figure 4A, a significant decrease in intraregional FC values was observed in the low score group in ROIs in the i-PFC (*t* = 5.444, *p* < 0.001), i-PSMA (*t* = 3.739, *p* = 0.002), i-PSC (*t* = 2.671, *p* = 0.017), i-M1 (*t* = 2.671, *p* = 0.017), c-PSMA (*t* = 3.836, *p* = 0.002) and c-OL (*t* = 3.018, *p* = 0.009). For the high score group, the intraregional FC of i-M1 (*t* = 3.823, *p* = 0.002) was significantly decreased in the task state compared with the resting state. The results of correlation analysis show a significant negative correlation between FMA-UE scores and task-evoked decreases in intraregional FC of i-PSC (*r* = −0.564, *p* = 0.045) in the low score group and i-M1 (*r* = −0.612, *p* = 0.026) in the high score group, as shown in Figure 4B.

### RAT-related changes in HAI values in patients with different degrees of motor impairment

**Figure 5:**
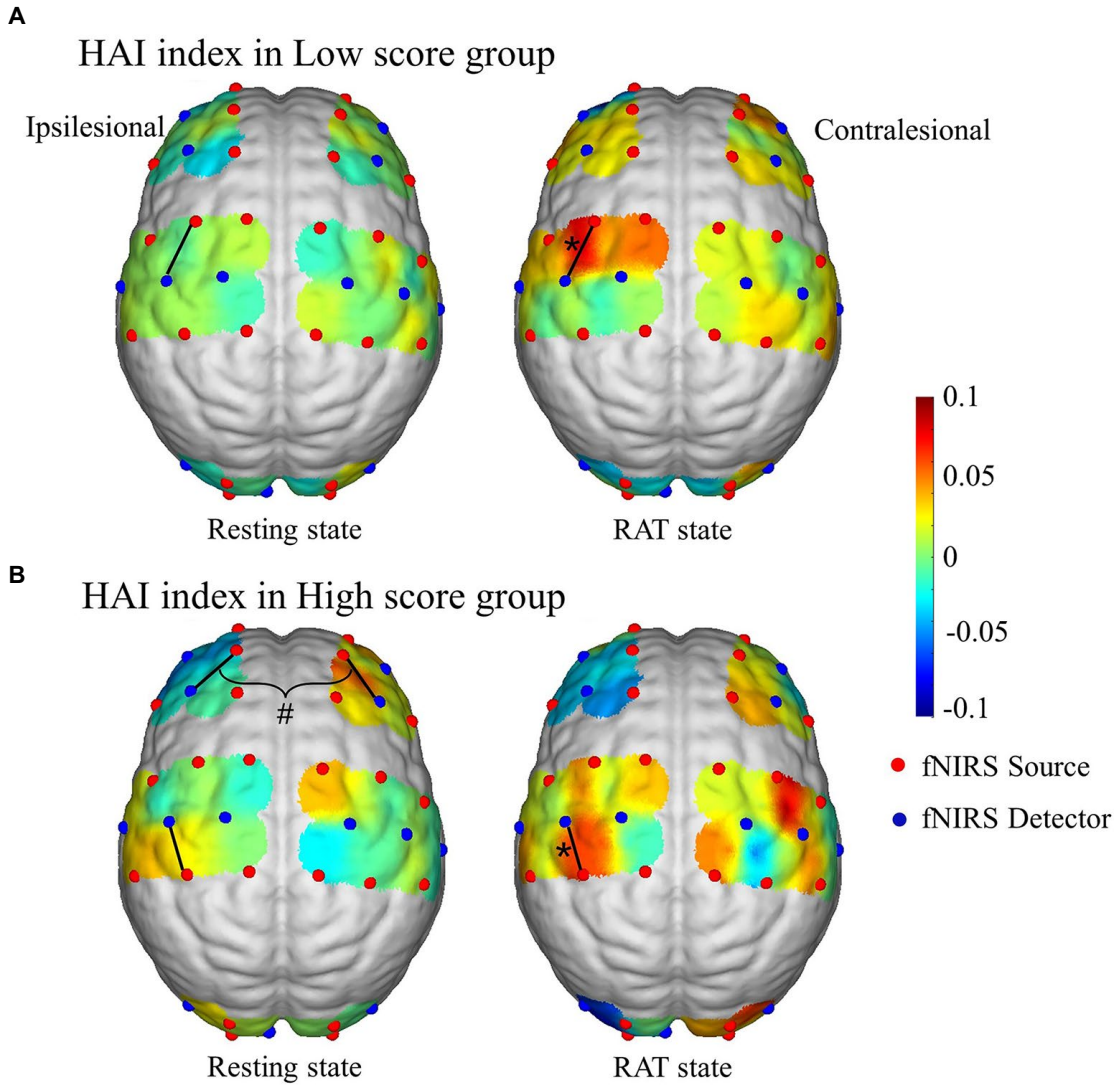
Connection-based HAI values in the resting state and RAT state in the high score group **(A)** and low score group **(B)**. *Denotes that the within-group difference is statistically significant; # denotes that the difference between hemispheres is statistically significant.

Figure 5 shows the connection-based HAI values in the resting state and RAT state for the two groups. The results show that compared with the resting state, the HAI values of channel 15 (*Z* = −2.497, *p* = 0.012) in the low score group and HAI values of channel 20 (*Z* = −2.497, *p* = 0.005) in the high score group were significantly increased in the RAT state. There were no significant differences between groups after correction. In addition, a significant difference in the bilateral hemisphere between channel 5 and channel 10 (*Z* = −2.844, *p* = 0.007) was observed in the resting state of the high score group.

## Discussion

The goal of this study was to investigate the differences in RAT-related brain functional responses in stroke patients with different degrees of upper-limb motor impairment. Specifically, we analysed the task-related changes in interregional and intraregional FC and the brain lateralization index of the functional network based on fNIRS. The main findings were that patients with severe impairment showed a wide range of significant FC responses induced by RAT with the same assistive mode as the moderate impairment group, involving the interregional and intraregional FC among bilateral prefrontal, motor and occipital areas. The significant task-related intraregional FC response of i-PSC was significantly correlated with FMA-UE in the low score group. Additionally, the HAI value of channel 15 distributed in the i-PSMA areas was significantly increased in the RAT state compared with the resting state. RAT-related extensive cortical response in patients with severe dysfunction might contribute to brain functional organization during motor performance, which is considered the basic neural substrate of motor-related processes. In contrast, the limited cortical response related to RAT in patients with moderate dysfunction might imply that the RAT task with assisted mode failed to induce wide range of brain functional responses and the training intensity needs to be adjusted in time according to the brain functional state for patients with moderate motor impairment. All the above evidence indicates that different functional response patterns associated with RAT depend on the degree of impairment. Real-time characterization of the brain function responses to specific training tasks is important for the assessment of functional status of stroke patients and provide guidance for the customization of effective rehabilitation training protocol.

Functional recovery after stroke is widely considered to be a consequence of central nervous system reorganization (Ward, 2004). In this study, we found that RAT substantially affected the functional networks of stroke patients by decreasing intrinsic network FC. This was evidenced in both the interregional and the intraregional FC. Task-related changes in FC play an important role in dynamically reshaping brain network organization and strongly contributing to brain activations during task performance (Cole et al., 2021). Compared with the resting state, there were significant changes in the interregional and intraregional FC values among the bilateral prefrontal, motor-related and occipital areas in the task state in the low score group. Performing complex motor tasks assisted by the robot system may require a higher level of attention and sensor-motor processing to integrate visual, proprioceptive, and somatosensory feedback information associated with motor output (Betti et al., 2013; Spadone et al., 2015; Kim et al., 2018). However, only the connectivities between the i-M1 and the i-PFC, between the i-M1 and the c-PSC, and between the i-M1 and the c-M1 showed significant task-related changes in the patients in the high score group. This result suggests that patients in the low-score group need to recruit a wider range of brain regions to complete the same motor task than patients in the high-score group.

Task-related network reconfigurations might facilitate the propagation of task-related activations, which are commonly considered the primary neural substrate of motor execution processes (Cole et al., 2021). For patients with severe motor impairment, the significant alterations in the interregional and intraregional FC involved bilateral hemispheres might be responsible for processing and integrating the central and peripheral information related to the task demands (Siegel et al., 2016). It has been confirmed that movement of the affected hand is related to increased neural activity not only in the ipsilesional but also in the contralesional hemisphere (Chollet et al., 1991). Additionally, it is suggested that when the task becomes more demanding, motor performance depends more on bilateral motor areas (Verstynen and Ivry, 2011). Motor recovery has been demonstrated to be accompanied by increased regional cerebral blood flow in the bihemispheric sensorimotor cortex (Chollet et al., 1991). All this evidence suggested that the contralesional motor areas might play a supportive role during motor rehabilitation for patients with severe motor impairment. In addition, correlation analysis showed that the task-induced changes in the functional network of the ipsilesional sensory area were significantly correlated with the upper-limb motor function status of patients with severe motor impairment. Brain lateralization analysis showed that the HAI values of fNIRS channels (ch13-ch17) covering the ipsilesional pre-motor and supplementary motor area (SMA) were increased in the RAT state compared with the resting state, which were significantly increased in the channel 15. More specifically, the location of channel 15 distributed in the ipsilesional SMA. Hemisphere lateralization is a property of the human brain that facilitates efficient and rapid information processing. The HAI can reflect cortical functional lateralization based on the imbalance of intrahemispheric and interhemispheric connectivity (Wang et al., 2014). This result might indicate the increased involvement of the ipsilesional SMA area in the brain functional network during RAT of the affected upper limb in patients with severe motor impairment. It was suggested that motor improvement in stroke is associated with cortical function and structural reorganization involving the lesion and its surrounding tissue (Buch et al., 2016). A previous study showed that functional improvement of constraint-induced movement of the affected upper limb after stroke is associated with an increased motor map area in the ipsilesional hemisphere (Sawaki et al., 2008). Taken together, all this evidence indicates that patients with severe motor dysfunction significantly induced involvement of the contralesional hemisphere and the sensorimotor and supplementary motor areas on the ipsilesional side during the RAT in assisted mode. This finding was in accord with previous research that showing high-intensity upper limb training in the early stage of rehabilitation can increase the activation of the motor areas in the ipsilesional hemisphere and enhance neuroplasticity (Zhang et al., 2017). The upper limb rehabilitation robot can control the expansion of the contralateral (the opposite side of hemiplegic limb) cortical motor area and the recruitment of the ipsilateral (the same side of hemiplegic limb) cortex through task-directed training, and promote the functional reorganization of the functional cortex to promote functional recovery of the upper limb (Singh et al., 2021).

Patients in the high score group showed limited task-related significant changes in the functional network, which were mainly related to the M1 region on the affected side. There was a significant correlation between the FMA-UE and the intraregional FC of the i-M1 in the high score group. In addition, the HAI value of channel 20 distributed in the ipsilesional M1 was significantly increased in the RAT task state compared with the resting state in the high score group. In conclusion, the brain functional responses induced by the RAT task mainly focused on the ipsilesional M1 area for patients with moderate impairment. The above point of view and our results might imply that compared with the outcome for the low score group, the RAT task with assisted mode failed to induce wide range of brain functional responses for patients with moderate motor impairment. These results might indicate that the mode of motor training rehabilitation needs to be adjusted in real time according to the functional status of patients to ensure that an adequate brain functional reorganization response can be induced.

## Limitations

Several limitations should be acknowledged in this study. First, stroke patients with moderate to serve upper extremity impairment in the subacute stage were recruited in the current experiment. The lack of a subgroup of stroke patients with mild motor impairment and controlled group based on healthy subjects or RAT with unaffected upper-limb of stroke patients is a potential limitation of this study. Future experimental design would recruit more stroke patients with different degrees of motor impairment (including mild, moderated and severe dysfunction) and the healthy controls to fully describe the characteristics of the brain functional response related to specific rehabilitation tasks. Second, due to the difference of cortical activation patterns related to active and passive upper limbs movements in stroke patients (Xia et al., 2022), the influence of active participation on brain functional response in the motor training should be considered. In this study, “assisted as needed” pattern of RAT was set uniformly for patients in the subacute stage with severe–moderate upper limb motor impairment. Under this training protocol, robotic device might provide more assistance to the patients with severe motor impairment than the patients with moderate motor impairment. However, due to the lack of kinematical variables in this study, it is difficult to estimate the influence of active participation degree of stroke patients with different degrees of dysfunction on the cortical response during assisted training. Thus, further studies are warranted to clarify the influence of the active participant on the cortical response induced by RAT by the collection of kinematic data simultaneously.

## Conclusion

In conclusion, this study showed RAT-related changes of decreased intrinsic network FC in the brain functional networks of stroke patients, with evidence in both the interregional and the intraregional FC. Different functional responses related to RAT were observed in patients with different degrees of dysfunction. Patients with severe motor impairment showed a significant task-related FC response involving extensive areas in the bilateral hemispheres, especially the PSC and SMA in the affected side. The brain functional responses induced by the RAT task mainly focused on the ipsilesional M1 area for patients with moderate impairment. The limited cortical response related to RAT in patients with moderate dysfunction might imply that the RAT task with assisted mode failed to induce wide range of brain functional responses and the training intensity needs to be adjusted in time according to the brain functional state for patients with moderate motor impairment. Taken together, fNIRS-based real-time assessment of the effects of RAT on the brain functional network provides new insights into the mechanisms of neuroplasticity associated with treatment and provides theoretical guidance for stroke rehabilitation intervention protocol.

## Data availability statement

The data that support the findings of this study is available from the corresponding author upon reasonable request.

## Ethics statement

The studies involving human participants were reviewed and approved by Medical Ethics Committee of Qilu Hospital. The patients/participants provided their written informed consent to participate in this study.

## Author contributions

CH, ZS, XL, HX, and YS: data collection and investigation. CH, HX, and GX: data analysis. CH: manuscript draft. ZS: manuscript revision. YW and ZL: supervision. All authors contributed to the article and approved the submitted version.

## Funding

The research is supported by the National Natural Science Foundation of China (NSFC Numbers 81672249, 81972154, 32271370, and 82172536). National Key Research and Development Project No. (Grant No. 2020YFC2004200) and the Fundamental Research Funds for Central Public Welfare Research Institutes (118009001000160001).

## Conflict of interest

The authors declare that the research was conducted in the absence of any commercial or financial relationships that could be construed as a potential conflict of interest.

## Publisher’s note

All claims expressed in this article are solely those of the authors and do not necessarily represent those of their affiliated organizations, or those of the publisher, the editors and the reviewers. Any product that may be evaluated in this article, or claim that may be made by its manufacturer, is not guaranteed or endorsed by the publisher.
